# Annotations

**Published:** 1889-09-28

**Authors:** 


### Annotations.

**All the World Over.**

Persons with eyes and ears who do not presume to call themselves by the portentous name of social philosophers, gradually but surely come to the conclusion that human nature is much the same all the world over. We do not look to annual reports of charitable societies for exciting reading. But even from these it is possible to get a good deal of light, and sometimes not a little comfort. The light that reveals the fact of a shiftless class in every part of the globe, consoles us with the assurance that we are not worse off in this respect than our neighbours, and, moreover, gives the justifiable hope that the majority of those who are not shiftless manage to do fairly well in every country. The shiftless sink to the bottom of the social ocean by their own inertia and weight. When we see them all massed together at the bottom we think how dreadful it is. We forget that they are the sinkings of the whole body social, and that the upper layers are almost entirely self-supporting and fairly happy. It is to be remembered that poverty and want are Nature's whips whereby she tries to make the idle industrious, and the shiftless capable. It is neither wise nor right to interfere too much with her wholesome discipline. The current report of the Associated Charities of Columbia, which was read to the annual meeting held at New York a short time ago, describes the efforts made by Congress and by charitable individuals to stem the tide of misery, and the fruitlessness of those efforts until thorough investigation and systematic relief on definite lines were introduced. It was found that relief given without adequate investigation "encouraged and multiplied beggars, many of whom were able to earn their own living, but preferred to patronise the free or penny lunch houses, and resorted to gross deception to obtain food and fuel for themselves and their families, either by being first at the precinct station, or the most zealous in inducing some sympathetic friend to intercede in their behalf to make theirs a special case with the police or with an agent of the Provident Society, generally receiving from both, and perhaps from churches and persons privately at the same time." It reads exactly as it would have done in London. The born beggar will beg, and will not work, happen what may. Then he should starve. "There are multitudes of honest, suffering poor," continues the report, "and those most meritorious are generally the last to let their wants be known." That is entirely the case at home. The honest and independent-minded will rather starve than beg, and they are to be commended for it. Pauperism is hateful, and should never be covered with a pleasing mask. "Investigate daily for years" is the note of the Associated Charities, then "you have learned something of human nature as it is." Undoubtedly! It cannot be too often repeated that indiscriminate almsgiving is the soil in which idle and vicious paupers spring up and thrive. So long as that continues, so long will there be all the world over a huge class who produce nothing and suck the life-blood of others. It will pay the State, and all kinds of administrators of public and private charities, to make it a standing rule to disburse nothing, even of the value of a rhubarb powder, without investigation.

**Hypnotism and Theosophy.**

The subject of theosophy has not much attraction for the ordinary mind. The very name suggests to the average Briton delusion and chicanery, and it is quite possible that the average Briton who went to Colonel Olcott's lecture on the subject on the 17th, was a little disappointed to find there so little that was mystical and extravagant. But to many the most interesting of his remarks were those which referred to hypnotism. Hypnotism has certainly shaken the materialism of the modern mind. Just when we had comfortably settled down to the belief that we knew all about everything, there comes Charcot with his experiments at the Salpêtrière, and Messrs. Binet and Féret with their volume on Animal Magnetism, to show us that there are things in heaven and earth not dreamed of in our philosophy. We may each indulge in our own theory as to how the phenomena of hypnotism are brought about, but it is impossible to deny their existence. Simultaneously we learn that these phenomena were observed by long-dead Eastern sages. They were produced at will by the Charcots of prehistoric ages, and are produced at will by their descendants to-day. Charcot has somehow laid his finger on one of the hitherto unknown forces of nature, and has thereby—most unwittingly it may be—confirmed the pretensions of those who have hitherto been regarded as deluded dreamers or unscrupulous charlatans. These dreamers or charlatans claim to possess a greater control of these forces than any of our scientists; but it is no longer possible to pooh-pooh them so easily. The most sceptical among us admit that there may be something in it. Meanwhile, hypnotism pays the penalty of popularity by becoming an after-dinner experiment—a parlour game. This, Colonel Olcott condemns strongly, and with justice. Where the "thought-reading," the "willing," and the rest fail, it does not much matter. But when they succeed, the weaker nature must obey the will of the stronger, and a slavery is established which becomes daily more complete and pernicious. Such experiments, leading to such a slavery, are, says Colonel Olcott, "dangerous and even criminal." It was a wise restriction on the part of the old Indian magi, that kept their knowledge a thing confined to few, and not easily attained. If the power which one mind can exercise over the mind and body of another were measured and known, it might be less serious to meddle with it; but considering the delicacy of the instrument played upon, and our present imperfect knowledge of the laws which control it, experiments in hypnotism must be regarded as equally dangerous with experiments with explosives or poisons.

**The Luxury of Fresh Eggs.**

Is there one well-conditioned man in a hundred who will demur to the statement that, among the minor luxuries of life, an abundance of fresh eggs for the table is one of the chief? The sight of the curdling cream of a quite new-laid egg when the shell is cracked is one of the finest breakfast appetisers known to medical science. And, *per contra*, the sight and smell of a month old "Shop 'un" as "Middlewick" has it, makes breakfast an impossibility to some and a disgust to all. In a morning contemporary there has lately been carried on an interesting correspondence about fowl-keeping in general, and the cost of chickens and new-laid eggs in particular. Mr. Tegetmeier and Mr. Bartlett, it appears, have each given it as his independent dictum that fowl-keeping on a large scale does not pay. In the columns of our contemporary this morning (September 20) there are no fewer than nine separate letter writers, each of whom declares his conviction that it does pay; whilst, on the side of the unbelievers, there is not a solitary line of any kind. It has been our fortune to have experience of fowl-keeping on both a large and a small scale, and we have not the slightest hesitation in giving our vote on the side of the believers in fowls. Of course, a man who keeps a hundred fowls need not expect to make £100 a year of profit by them. And that is probably where the shoe pinches. Fowls undoubtedly give a good deal of trouble; and if the average Briton takes trouble, he likes to be well paid for it. That, we believe, is the whole secret of the scepticism of Fowl Sceptics; and, indeed, when one thinks of it, it is the whole secret of the scepticism of all sorts of sceptics. They have such a mighty opinion of their own overwhelming merits, that no amount of profit or advantage can satisfy their expectations. A fresh egg of a good size, when compared with other albuminous foods in value and price, is worth twopence all the year round. At a penny or threehalfpence, it is one of the cheapest foods that can be purchased both for grown up people and children. It can be cooked in a hundred different ways. Moreover, there is no doubt whatever that, even at a penny an egg, all the year round, a price which there is no difficulty in obtaining, there is at least 25 per cent. of profit on actual outlay, if the labour of the caretaker be not included. Now, the question is, do hens require so much care and time to be spent upon them as to make 25 per cent. of gain on outlay finally unprofitable? We say they do not. To many people, indeed, the care of a dozen or a score of hens is much more of a pleasure than of a trouble, and is decidedly advantageous to health. If the advantage to health be taken in conjunction with the benefit arising from the eating of plenty of perfectly fresh eggs, there cannot be the slightest doubt, at any rate from a medical point of view, about the profitableness of keeping fowls. People who have a little bit of garden, and a modicum of spare time, can hardly employ it better than by developing a well-kept fowlrun.

